# Geographical variation of unmet medical needs in Italy: a multivariate logistic regression analysis

**DOI:** 10.1186/1476-072X-12-27

**Published:** 2013-05-12

**Authors:** Marina Cavalieri

**Affiliations:** 1 Department of Economic and Business, University of Catania, Corso Italia 55, Catania 95129, Italy

**Keywords:** Italy, Unmet health care needs, Access to health care, Barriers to health care, Decentralization

## Abstract

**Background:**

Unmet health needs should be, in theory, a minor issue in Italy where a publicly funded and universally accessible health system exists. This, however, does not seem to be the case. Moreover, in the last two decades responsibilities for health care have been progressively decentralized to regional governments, which have differently organized health service delivery within their territories. Regional decision-making has affected the use of health care services, further increasing the existing geographical disparities in the access to care across the country. This study aims at comparing self-perceived unmet needs across Italian regions and assessing how the reported reasons - grouped into the categories of availability, accessibility and acceptability – vary geographically.

**Methods:**

Data from the 2006 Italian component of the European Union Statistics on Income and Living Conditions are employed to explore reasons and predictors of self-reported unmet medical needs among 45,175 Italian respondents aged 18 and over. Multivariate logistic regression models are used to determine adjusted rates for overall unmet medical needs and for each of the three categories of reasons.

**Results:**

Results show that, overall, 6.9% of the Italian population stated having experienced at least one unmet medical need during the last 12 months. The unadjusted rates vary markedly across regions, thus resulting in a clear-cut north–south divide (4.6% in the North-East vs. 10.6% in the South). Among those reporting unmet medical needs, the leading reason was problems of accessibility related to cost or transportation (45.5%), followed by acceptability (26.4%) and availability due to the presence of too long waiting lists (21.4%). In the South, more than one out of two individuals with an unmet need refrained from seeing a physician due to economic reasons. In the northern regions, working and family responsibilities contribute relatively more to the underutilization of medical services. Logistic regression results suggest that some population groups are more vulnerable than others to experiencing unmet health needs and to reporting some categories of reasons. Adjusting for the predictors resulted in very few changes in the rank order of macro-area rates.

**Conclusions:**

Policies to address unmet health care needs should adopt a multidimensional approach and be tailored so as to consider such geographical heterogeneities.

## Background

Though the Italian National Health Service (*Servizio Sanitario Nazionale*, *SSN*) is grounded on the Constitutional principles of universalism and comprehensiveness, many Italians still perceive that they do not receive the health care they require. One commonly used indicator of access to care is self-assessed unmet health care need. Compared to the prevailing approach in measuring access to health care, which is based on actual utilization (e.g. physician visits and hospitalization rates), the use of subjective indicators allows not only to account for those perceived medical needs that do not turn into demand, but also to investigate the process of seeking medical care and the subjective barriers that individuals with health needs encounter in accessing it.

Defining an unmet health care need is, however, not an easy task. According to Carr and Wolf [1], an unmet need is “the differences, if any, between those services judged necessary to deal appropriately with defined health problems and those services actually being received … an unmet need is the absence of any, or of sufficient, or of appropriate care and services”. Consistent with this definition, only those clinically assessed needs which are not satisfied by appropriate health care can be considered unmet. In this respect, the subjective assessment of unmet health needs diverges from the above definition, since it has the potential to detect subjective unmet health expectations which are not necessarily clinically grounded. Conversely, it neglects unperceived (but clinically relevant) unmet health needs [2]. Notwithstanding this, the latter approach for measuring unmet needs is considered superior from several points of view. First of all, it is more feasible since standardized questions on unmet needs for health care are nowadays included in most periodically conducted national surveys. Secondly, the subjective assessment of unmet needs is consistent with the assumption that, due to asymmetric information problems, the patient is the best judge of his/her health status and of whether he/she has received appropriate health care.

Research on self-perceived unmet health care needs has been carried out mainly in the US [3-6] and Canada [7-13]. As a general trend, a rise in the proportion of people reporting unmet health needs over time is detected [8]. Although one should be aware about making comparisons between studies because of the variability in the methods and designs used, rates of unmet health needs in the US are generally found to be higher (between 5% and 20%) than those in Canada (between 4% and 12%), thus showing that unmet health needs are strongly influenced by the extent of health insurance coverage. Other specific vulnerable sub-populations (e.g. women, less healthier and low-income individuals, immigrants, etc.) are found to be more likely to experience problems in meeting their health needs [4-7,9-12].

A small group of studies from the above mentioned countries has explicitly addressed the question of whether individuals who report an unmet need are also using more health services than would be expected on the basis of their need [2,5,14,15]. These found evidence of a systematic positive association between certain typologies of reported unmet health care needs and specific utilization patterns, after controlling for needs.

As for Europe, few papers have empirically explored the issue of unmet health care needs. Two Swedish studies have analysed variations in unmet health needs across population groups [16,17]. Comparisons across European countries in self-reported unmet health needs have been made possible by the existence of two international surveys: the Survey on Health, Ageing and Retirement in Europe (SHARE) and the European Union Statistics of Income and Living Conditions (EU-SILC) [18-20].

To the best of our knowledge, no previous published studies have analysed unmet health care needs in Italy. This study intends to fill the existing gap in the literature by examining regional disparities in the the rates of self-perceived unmet medical needs and in the reported reasons. The study also investigates how demographic, socio-economic and health status variables may contribute to explain the results.

The analysis of unmet medical needs at the regional level is particularly important in Italy where a progressive decentralisation process has been taking place since the 1990s, increasing the powers of Regional Health Authorities in both the financing and delivery of health care. Regions are now fully responsible to organize their health care systems so as to deliver the essential levels of care (*Livelli Essenziali di Assistenza, LEA*) which must be guaranteed nationwide. However, marked organizational differences exist in the way Regions have decided to accomplish their health delivering task, which could potentially result in different access rates and barriers to health services.

The significance of this paper is, however, not limited to the Italian context. The use of EU-SILC data makes this research approach interesting to other European member states. The study’s objective is of particular importance for those European countries (e.g. Denmark, Portugal, Spain, Sweden, etc.) that rely on a National Health Service but have decided to decentralize health responsibilities to lower levels of governments. For these countries, the existence of intra-country differences in unmet health needs and in the related reasons, further strengthen the choice to decentralize health care so as to better tailor health policy responses to local specificities. Outside Europe, other countries (e.g. Canada) with similar health system characteristics may show a similar interest in the empirical approach of this work. Finally, also for those countries where the health care system is highly centralized, it can be useful, for policy purposes, to investigate whether geographical differences exist in the prevalence of unmet needs and in the reported reasons.

From a methodological point of view, compared to cross-country analyses, sub-national ones require less cautions in formulating conclusions, since unmeasured heterogeneity in the dataset due to cultural and health system differences can be better controlled for.

### The Italian health care system

Italy has a National Health Service which was established in 1978 to provide comprehensive health insurance coverage and uniform health benefits to all citizens and legal residents, throughout the country. The SSN is funded from general taxation and organized on the basis of a three-tier structure of government: central (Ministry of Health), regional (20 Regional Health Authorities) and local (*Aziende Sanitarie Locali* – *ASLs*). The actual SSN organizational structure is the result of a set of reforms undertaken since the early 1990s, aimed at introducing quasi-market mechanisms into the health system as well as at devolving new responsibilities for both the financing and delivering of health care to regions.

Alike in other countries (e.g. Denmark, Spain, Sweden, Switzerland, Canada, etc.), decentralization of health care has been seen as a way to improve local responsiveness, promote participatory democracy, contain health expenditure and enhance efficiency. Regions are free to organize their health care services according to their local specificities but it is the central government’s responsibility to define and monitor the LEA services.

In Italy, primary care is provided by independent contracted general practitioners (GPs) and pediatricians paid on a capitation basis that act as gatekeepers for the access to secondary services. Individuals may choose any physician they prefer, provided that the physician’s list has not reached the maximum number of allowed patients (i.e. 1,500 for GPs and 800 for pediatricians).

Specialized ambulatory services, including visits and diagnostic and curative activities, are provided either by ASLs or by other public and accredited private facilities with which ASLs have made agreements and signed contracts. Patients are allowed to access specialist care only after approval by their GP, who is responsible for the referral. Once the GP has authorized the visit or the procedure, patients are free to choose any provider among those accredited by the SSN and any place of treatment.

Depending on the region of residence, the payment of a user charge (called “ticket”) is sometimes required as an additional source of financing and in an attempt to discourage an inappropriate use. Services for which a co-payment is required include ambulatory treatments, diagnostic and laboratory tests, specialist care, drugs, medical appliances and glasses. Inpatient care and primary care are free at the point of use. In emergency cases, direct and free access is allowed for all health services. Nonetheless, cost-sharing exemptions are allowed for specific groups, depending on income, age, health conditions and other individual characteristics.

Because of long waiting lists and the not always satisfactory quality of public services (especially in the southern regions), many individuals often consult private outpatient specialists, at their own expense. As in other European countries (e.g. Greece, Portugal, Spain, etc.), with increasing personal income levels, individuals often opt to supplement their public health insurance with the purchase of private insurance and/or private services. Although the utilization of private health services differs greatly by region, in 2005 an average of about 5% of primary care visits, 57.2% of specialist visits and 21% of diagnostic services were entirely paid out of pocket by individuals [21].

## Methods

This study is based on data from the European Union Statistics on Income and Living Conditions (EU-SILC). Although the EU-SILC survey is mainly oriented to the analysis of poverty and deprivation, it also provides the opportunity to explore issues related to health accessibility: a set of items is specifically designed to measure self-assessed health status and barriers encountered by household members aged 16 and over in trying to access medical services.

The 2006 EU-SILC module for Italy, conducted by the National Institute of Statistics (*Istituto Nazionale di Statistica, ISTAT*) through face-to-face interviews, comprises 21,499 households (54,512 individuals) who are selected from across the country, using a stratified two-stage sampling design. We restricted our analysis to adults aged 18 and older (45,358 eligible observations) since access to health care services by individuals under that age is generally tied to the decisions of their parents or legal guardians.

Self-reported unmet medical need was defined on the basis of the following question: “During the last 12 months, was there ever a time when you felt you needed a visit by a specialist or a medical treatment but you did not receive it?”^1^. Respondents who replied affirmatively were coded as having an unmet need and were then asked the main reason for not getting medical care. Possible answers were: 1) could not afford to (too expensive); 2) too long waiting lists; 3) could not take time because of work, care for children or for others 4) too far to travel/no means of transportation; 5) fear of doctor/treatment; 6) wanted to wait and see if the problem got better on its own; 7) did not know any good doctor or specialist; 8) other reasons. Multiple responses were not allowed.

Following the previous literature on access barriers to health care services, answers were classified into four categories according to the nature of the stated reason: availability, accessibility, acceptability and other [7]. The first group includes only the “waiting list” response, as an indicator of unavailability of the service at the time required. The accessibility category relates to barriers, such as financial and transportation problems, that are not voluntarily chosen by the individual and can be hardly overcome in the short run. With the exception of the “other” response which is separately tabulated, all the remaining reasons, partly due to personal choices and mainly concerning attitudes, personal beliefs and competing responsibilities, are grouped into the acceptability category.

In an attempt to better disentangle the potential role played by policy makers in reducing unmet health needs, some authors [15,20] have suggested to separate system-related (waiting times and costs) from personal (all remaining motives) reasons for unmet health needs. Behind this choice lies the view that, while unmet needs due to personal circumstances require little policy response (or at least interventions other than health system ones), those due to system-related reasons may be a symptom of a poorly performing health system which calls for active policy interventions by the central government and/or the regional authorities.

Potential predictors of unmet medical needs were selected according to the Andersen’s Behavioural Model of health service use [22]. The model assumes that a person’s use of health services is a function of *predisposing*, *enabling* and *need* factors. *Predisposing* factors include socio-demographic variables and health beliefs that influence the propensity of individuals to seek health care. The *predisposing* variables used in this paper are age, sex, level of education, marital and activity status. *Enabling* factors include personal, family and community resources that can either facilitate or impede the use of health services. Hence, individuals were grouped into quintiles according to their equivalised annual disposable (i.e. after-tax) household income. The modified OECD equivalence scale was used, which gives a weight of 1.0 to the first adult in the household, 0.5 to the second and each subsequent person aged 14 and over, and 0.3 to each child aged under 14. Under the EU-SILC, household total disposable income includes all of the net monetary income received by the household and its members during the reference year - namely all income from work (employee wages and self-employment earnings), private income from investment and property, transfers between households plus all social transfers received directly including old-age pensions, net of any taxes and social contributions paid. No account is taken of indirect social transfers. Three measures of *need* are used in this study: 1) self-rated general health status (five-point scale from very good to very bad); 2) the presence of chronic conditions; 3) the presence of limitations in daily activities due to health problems.

We entered all of the above explanatory variables into multivariate logistic regression models for unmet needs overall and for the different typologies of reasons. Least squared means resulting from the models were used to determine adjusted rates. To simplify the sub-national analysis, the nineteen Italian regions and the two autonomous provinces were grouped into five macro-areas: North-West, North-East, Centre, South and Islands. Data were also weighted at individual level (cross-sectional weights provided by the EU-SILC) to make the results representative for the Italian general population. Missing and partial information accounted for less than 0.5% of the total and were therefore dropped from the final sample (45,175 respondents representing the population of about 48.8 million). Robust estimators of variance that accounted for the effects of weighting were used [23-25].

## Results

**Figure 1:**
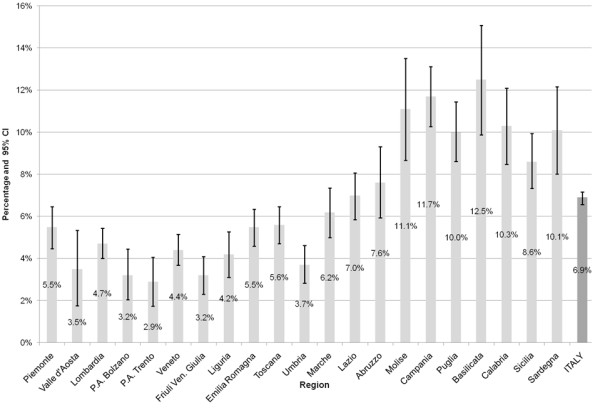
**Percentages of individuals reporting any unmet medical need by region (and 95% confidence intervals).** Note: Weighted sample used.

In 2006, approximately 6.9% of the Italian population aged 18 and older experienced at least one unmet need for health care over the past 12 months (Figure 1). The rate of self-perceived unmet need by region varied from 2.9% in the Province of Trento to 12.5% in Basilicata.

The analysis by geographical macro-area provides better insights into the existence of a north–south divide (Table 1). In the northern and central parts of Italy the percentages of people with unmet medical needs were significantly lower than the national average, with the minimum value observed in the North-East (4.6%). On the contrary, the percentage of individuals with at least one unmet medical need reached its maximum in the southern part of the country where a value of 10.6% was found. In the two main islands (i.e. Sardegna and Sicilia), the rate of unmet needs was around 9%. As a measure of the intra-area variability of the regional percentages of people with unmet health needs, coefficients of variation (CV) were also computed. Results show a greater variability of values in the North and a lower one in the South.

**Table 1:** Percentages of individuals reporting any unmet medical need by macro-area

**Macro-area**	**Regions**	**Percentage**	**SE**	**CI**	**CV**
North-West	Piemonte, Valle d’Aosta, Lombardia, Liguria	4.9	0.27	(4.3; 5.4)	4.42
North-East	Autonomous Provinces of Bolzano and Trento, Veneto, Friuli Venezia Giulia, Emilia Romagna	4.6	0.24	(4.1; 5.0)	4.57
Centre	Toscana, Umbria, Marche, Lazio	6.2	0.31	(5.6; 6.8)	3.91
South	Abruzzo, Molise, Campania, Puglia, Basilicata, Calabria	10.6	0.4	(9.8; 11.4)	2.90
Islands	Sicilia, Sardegna	9.0	0.56	(7.9; 10.1)	3.18
*ITALY*		*6.9*	*0.15*	*(6.6; 7.2)*	*3.68*

*Note*: All results are weighted. SE = robust standard errors. CI**=** 95% confidence intervals. CV= coefficients of variation. For each macro-area, the CV is computed as the ratio of the standard deviation of the regional percentages of population with unmet needs to the mean percentage in the macro-area.

**Figure 2:**
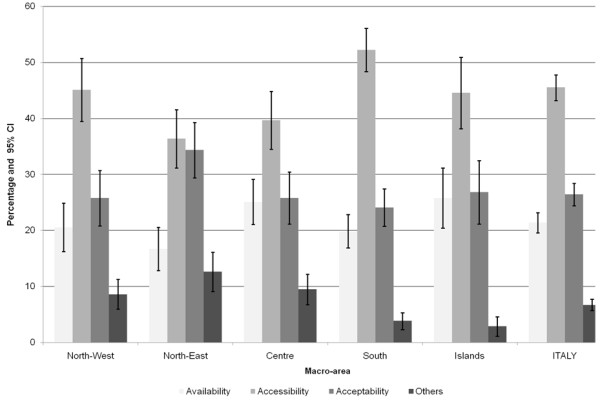
**Self-reported reasons for having perceived unmet medical needs, by macro-area (% and 95% confidence intervals).** Note: Weighted sample used. Multiple responses were not allowed.

Figure 2 presents the percent distribution of reasons (by category) behind unmet medical needs by macro-area. Availability-related unmet medical needs due to the existence of too long waiting lists accounted for 21.4% of all the reasons reported at a national level. They were more likely in the Islands (25.8%) and in the Centre (25.1%) than in the North-East (16.7%). Waiting lists were a minor concern in the Autonomous Province of Bolzano where only 4.2% of the individuals reported them as the reason for their unmet medical needs. Accessibility issues were the most frequently reported reasons by the entire Italian population (45.5%). Among these, the percentage of cost-related unmet needs reached 44.4% at a national level while transportation difficulties were a minor barrier, identified by only 1.2% of the Italians with unmet medical needs. The analysis by macro-area shows that affordability was a serious problem especially in the poorer and less developed South, where more than one out of two individuals with an unmet need refrained from seeing a physician due to economic reasons. Working and family responsibilities contribute to an underutilization of medical services too. About 11% of those Italians who refrained from seeking a doctor despite a perceived need reported lack of time. Altogether, other acceptability problems such as fearing doctors, deciding not to bother or not knowing where to go accounted for approximately 15% of unmet needs at a national level. In the South and the Islands, it appeared to be less difficult to reconcile medical visits with work and family commitments (9% in both areas).

The weighted national sample was used to disentangle the role of the selected variables in reporting unmet medical needs overall and the three main categories of reasons (availability, accessibility and acceptability). Distribution of study population and self-perceived unmet medical needs by the predictor variables used in the multivariate logistic regression analyses are provided in Table 2. Table 3 displays the adjusted odds ratios from the logistic regression models.

**Table 2:** Distribution of study population and self-perceived unmet medical needs by the selected variables (% and 95% confidence intervals)

**Variable**	**Study population**	**Population reporting unmet needs**
***Predisposing factors***		
*Sex*		
Male	51.9 [51.3-52.5]	5.9 [5.5-6.3]
Female	45.0 [44.4-45.6]	7.7 [7.3-8.2]
*Age*		
18-44	48.1 [47.5-48.7]	5.8 [5.4-6.2]
45-64	30.7 [30.2-31.2]	7.7 [7.2-8.3]
65+	24.3 [23.9-24.8]	7.7 [7.1-8.3]
*Education*		
Primary	56.0 [55.4-56.6]	8.0 [7.6-8.4]
Secondary	33.5 [32.9-34.0]	5.6 [5.2-6.1]
Tertiary	10.5 [10.1-10.9]	4.6 [3.8-5.4]
*Marital status*		
Married	58.6 [58.0-59.2]	6.9 [6.5-7.2]
Unmarried	28.0 [27.4-28.5]	5.2 [4.7-5.7]
Separated/divorced/widowed.	13.5 [13.1-13-9]	10.4 [9.4-11.3]
*Employment status*		
Currently working	54.4 [53.8-55.0]	7.6 [7.1-8.0]
Other (unempl., retired, student, econom. inactive, etc.)	45.6 [45.0-46.2]	6.0 [5.6-6.4]
***Enabling factors***		
*Equivalised Income quintile*		
0-20%	22.0 [21.5-22.5]	11.3 [10.5-12.2]
20-40%	19.8 [19.3-20.3]	7.2 [6.5-7.9]
40-60%	19.3 [18.9-19.8]	5.8 [5.2-6.4]
60-80%	19.0 [18.5-19.4]	4.9 [4.3-5.4]
80-100%	19.9 [19.4-20.3]	4.5 [4.0-5.1]
***Need factors***		
*Health status*		
very good	13.1 [12.7-13.5]	3.0 [2.5-3.6]
good	43.5 [43.0-44.1]	4.3 [3.9-4.6]
fair	32.8 [32.3-33.4]	9.5 [8.9-10.1]
bad	8.7 [8.3-9.0]	13.1 [11.8-14.5]
very bad	1.9 [1.7-2.0]	18.6 [15.2-22.0]
*Suffering from any chronic condition*		
No chronic	78.5 [78.0-79.0]	5.4 [5.1-5.7]
Chronic	21.5 [21.1-22.0]	12.0 [11.2-12.8]
*Limitation in daily activities*		
No limited	76.9 [76.4-77.4]	4.7 [4.4-5.0]
Limited	23.1 [22.6-23.5]	13.9 [13.1-14.8]
***Geographical macro-area***		
North-West	26.9 [26.4-27-5]	4.9 [4.3-5.4]
North-East	19.1 [18.7-.19.5]	4.6 [4.1; 5.0]
Centre	19.5 [19.1-20.0]	6.2 [5.6; 6.8]
South	23.3 [22.7-23.8]	10.6 [9.8; 11.4]
Islands	11.2 [10.7-11.6]	9.0 [7.9; 10.1]

*Note*: Weighted sample. Because of rounding, detail may not add to totals.

**Table 3:** Multivariate logistic regression models for overall medical unmet needs and the three categories of reasons

**Variable**	**Overall**	**Availability**	**Accessibility**	**Aceptability**
*Sex*				
Male	1.0	1.0	1.0	1.0
Female	1.20 [1.08-1.33]^***^	1.06 [0.86-1.3]	1.23 [1.05-1.45]^**^	1.23 [1.02-1.49]^**^
*Age*				
18-44	1.0	1.0	1.0	1.0
45-64	0.84 [0.73-0.97]^**^	1.14 [0.87-1.51]	0.80 [0.65-0.98]^**^	0.69 [0.53-0.88]^***^
*65+*	0.48 [0.40-0.57]^***^	1.20 [0.86-1.68]	0.28	0.57 [0.40-0.83]^***^
*Education*				
Primary	1.0	1.0	1.0	1.0
Secondary	1.03 [0.91-1.16]	0.99 [0.78-1.25]	0.95	1.17 [0.95-1.45]
Tertiary	0.91 [0 .74-1.13]	0.76 [0.49-1.16]	0.57 [0.37-0.88]^**^	1.34 [0.95-1.88]^*^
*Marital status*				
Married	1.0	1.0	1.0	1.0
Unmarried	0.88 [0 .77-1.01]^*^	1.11 [0.84-1.47]	0.75 [0.61-0.93]^***^	0.82 [0.63-1.06]
Separated/divorced/widowed	1.41 [1.22-1.62]^***^	1.23 [0.93-1.62]	1.57 [1.27-1.94]^***^	1.16 [0.88-1.53]
*Employment status*				
Currently working	1.36 [1.20-1.54]^***^	1.33 [1.04-1.69]^**^	1.14 [0.94-1.38]	1.82 [1.44-2.29]^***^
Other	1.0	1.0	1.0	1.0
*Equivalised income quintile*				
0-20%	1.0	1.0	1.0	1.0
20-40%	0.67 [0.58-0.77]^***^	0.91 [0.69-1.19]	0.52 [0.43-0.64]^***^	0.97 [0.75-1.27]
40-60%	0.57 [0.49-0.66]^***^	1.03 [0.77-1.37]	0.43 [0.34-0.53]^***^	0.78 [0.56-1.05]^*^
60-80%	0.51 [0.43-0.60]^***^	1.03 [0.76-1.41]	0.32 [0.25-0.41]^***^	0.77 [0.56-1.05]^*^
80-100%	0.53 [0.44-0.62]^***^	1.00 [0.73-1.36]	0.21 [0.15-0.30]^***^	1.05 [0.77-1.42]
*Health status*				
very good	1.0	1.0	1.0	1.0
good	1.52 [1.23-1.89]^***^	1.71 [1.09-2.66]^**^	1.19 [0.84-1.68]	1.88 [1.30-2.72]^***^
fair	3.04 [2.41-3.84]^***^	3.52 [2.21-5.62]^***^	2.46 [1.69-3.57]^***^	3.31 [2.24-4.91]^***^
bad	2.91 [2.20-3.85]^***^	2.57 [1.48-4.45]^***^	3.52 [2.27-5.43]^***^	1.79 [1.08-2.98]^**^
very bad	4.6 [3.26-6.48]^***^	3.28 [1.65-6.48]^***^	5.77 [3.49-9.55]^***^	1.40 [0.64-3.06]
*Suffering from any chronic condition*				
No chronic	1.0	1.0	1.0	1.0
Chronic	1.26 [1.11-1.44]^***^	1.06 [0.82-1.37]	1.44 [1.19-1.73]^***^	1.03 [0.79-1.34]
*Limitation in daily activities*				
No limited	1.0	1.0	1.0	1.0
Limited	2.21 [1.90-2.57]^***^	1.72 [1.31-2.26]^***^	2.21 [1.76-2.79]^***^	2.28 [1.70-3.05]^***^
*Geographical macro-area*				
North-West	1.0	1.0	1.0	1.0
North-East	0.91 [0.77-1.07]	0.74 [0.53-1.05]^*^	0.74 [0.57-0.95]^**^	1.21 [0.91-1.62]
Centre	1.23 [1.05-1.44]^***^	1.55 [1.16-2.07]^***^	1.02 [0.79-1.32]	1.26 [0.92-1.71]
South	2.0 [1.73-2.31]^***^	2.21 [1.66-2.94]^***^	1.78 [1.44-2.20]^***^	2.17 [1.64-2.87]^***^
Islands	1.61 [1.34-1.93]^***^	2.42 [1.70-3.43]^***^	1.19 [0.91-1.57]	2.07 [1.47-2.92]^***^

*Note*: All results are weighted. Adjusted odds ratios. Higher numbers indicate a greater odds of the outcome. 95% confidence intervals in parentheses.

^***^,^**^ and ^*^ indicate significance at 1%, 5% and 10%, respectively.

As for the predisposing factors, with the only exception of availability, women were statistically more likely to report unmet needs for each category of reasons. The likelihood of experiencing any type of unmet medical need decreased with age. Except for few cases, the level of education did not appear to be significantly associated with unmet needs. Unmarried individuals were less likely to report unmet needs overall and needs due to accessibility problems (especially financial ones), while the opposite seemed true for separated, divorced and widowed persons. Not surprisingly, activity status was found to be positively associated with the probability of remaining without care, especially because of not being able to take time off. Economically active people were much more likely to experience an unmet health need in general and to report an acceptability problem, in particular. With regard to the enabling factors, there was evidence of an income gradient. Other things being equal, individuals in the highest income quintile groups were less likely than those in the lowest ones to report unmet medical needs, particularly due to problems of accessibility. Health status showed a strong association with all of the reported reasons: people claiming to be in good or very good health were less likely to perceive any unmet medical need. Having any chronic condition also increased the likelihood of experiencing an unmet medical need, as did the fact of being hampered in daily activities because of health problems. Differences in the odds of unmet needs were observed among geographical areas. Independently of the stated reason, the odds of having experienced an unmet medical need were greater for people living in the South and the Islands than for those residing in the rest of the country. Except for the acceptability category, whose result was not statistically significant, people in the North-East had the lowest likelihood of reporting unmet medical needs.

Unadjusted and adjusted rates of self-perceived unmet medical needs, overall and by category of reasons are reported by macro-area and compared in Table 4. Adjusting for the predictors used in the logistic regression models resulted in few changes in the rank order. No changes were observed for overall unmet needs, though adjusted rates were always lower than the unadjusted ones. Regardless of the category of reasons, the North-East always maintained a constant ranking. As for unmet needs due to problems of availability, the two Islands were no more the leading regions in terms of rates: after adjusting for the selected variables, the Centre presented the highest percentage of unmet medical needs. Adjusted rates for accessibility-related unmet needs changed only mid-range rankings but did not influence the top (South) and bottom (North-East) positions. Finally, the lowest (adjusted) rates of unmet needs due to problems of acceptability were reported in the Centre and no longer in the South of Italy.

**Table 4:** Unadjusted and adjusted rates of self-perceived medical unmet needs, overall and by category of reasons, by macro-area (and rank order in parentheses)

**Macro area**	**Overall**		**Availability**		**Accessibility**		**Acceptability**	
	***Unadjusted***	***Adjusted***	***Unadjusted***	***Adjusted***	***Unadjusted***	***Adjusted***	***Unadjusted***	***Adjusted***
*North-West*	4.9 (4)	3.9 (4)	20.6 (3)	22.5 (3)	45.1 (2)	36.9 (3)	25.8 (4)	27.4 (4)
*North-East*	4.6 (5)	3.6 (5)	16.7 (5)	18.1 (5)	36.4 (5)	28.9 (5)	34.3 (1)	36.3 (1)
*Centre*	6.2 (3)	5.0 (3)	25.1 (2)	26.7 (1)	39.6 (4)	33.0 (4)	25.8 (3)	26.0 (5)
*South*	10.6 (1)	8.7 (1)	19.9 (4)	21.2 (4)	52.2 (1)	42.3 (1)	24.1 (5)	28.4 (3)
*Islands*	9.0 (2)	7.3 (2)	25.8 (1)	25.7 (2)	44.5 (3)	37.8 (2)	26.8 (2)	29.5 (2)

*Note*: All results are weighted.

Rates are adjusted for sex, age, education, marital status, employment status, equivalised income quintile, health status, chronic conditions, limitations in daily activities.

1 = highest rate of unmet needs; 5 = lowest rate of unmet needs.

## Discussion and conclusions

The empirical analysis suggests that, in spite of the SSN statutory obligation to provide equal access according to needs to all Italian citizens and legal residents - regardless of factors such as income, gender, ethnicity, education, religion and geographical location -, a certain percentage of the adult population claims that their health care needs are not met. This percentage is, however, not uniform across the country: there is evidence of marked regional variations in unmet needs overall and in the reported reasons. With very few exceptions, moving from the North to the South of the country, rates of self-perceived unmet medical needs increase and the stated reasons change. At the national level, cost of care is the most important reason reported for unmet health needs, followed by waiting lists and difficulties to reconcile the visits with work and family commitments. Consistent with its social and economic specificities, in the richer and more industrialized North competing priorities become a relatively more important motivation, albeit affordability is always the leading reason. On the contrary, in the South lack of financial means represents the sole relevant barrier to health need satisfaction. Here, work and family commitments are perceived as a minor problem, perhaps because of the lower employment rates, which make easier for individuals to take time off for seeking health care. Moreover, in these geographical areas a more extensive concept of family network exists, which helps women in their care-giving responsibilities, even in presence of worse-quality public services.

In line with previous international studies [7,9,10,12,13,16,18], logistic regression results suggest that some population groups are more vulnerable than others to experiencing unmet health needs and to reporting some categories of reasons. These include women, low income and less healthy individuals. Other individual characteristics are positively associated with unmet needs too: being young and separated/divorced/widowed, having a job, residing in the South and in the two main islands.

Some of the above findings require more in depth explanation. Gender differences in unmet needs can be due to the fact that women usually retain primary responsibilities as homemakers and family caregivers. These multiple roles generate more competing priorities and leave women with less time to seek care for themselves. However, the relationship between sex, health care use and unmet needs can also prove to work differently, though with the same final effect on unmet needs. Since women are more likely to be the primary care seekers for dependent children and elderly family members, they have generally more contacts with the health care system and, thus, more opportunities to experience difficulties in accessing care and, hence, to complain for unmet needs. As for age, prior studies suggest that expectations about the health care system may not be the same at different times in life [26-28]. In particular, younger patients tend to have higher expectations than elderly ones and thereby are more often dissatisfied with the care received. The positive association between activity status and unmet health needs can be explained by the fact that the former is expected to affect the time price of health care (i.e. the price of waiting time and travel time). Therefore, working individuals experience more difficulties to take time off for seeking care and, whenever they do it, the cost opportunity of their time is higher. Finally, as expected, income quintile is positively associated with reporting unmet needs due to problems of accessibility. The fact that the same variable is not a significant determinant of unmet needs due to long waiting times is not surprising. Indeed, it is consistent with a universal access single payer system, as the Italian one, where wealthy individuals cannot jump the queue to get necessary health care.

Adjusting for factors associated with unmet needs results in slight changes in the rank order by macro-area, which do not concern overall unmet medical needs but only the specific stated reasons. The dualism between the North and the South of the country persists. The residual variability across regions is likely to be explained by some organizational characteristics of the regional healthcare services (e.g. location and distribution of health services, co-payment decisions, etc.) that are not adequately captured by the determinants used in this analysis.

From a public policy perspective, these findings highlight the relative importance of system-related reasons (especially availability and accessibility ones), compared to personal motivations. To be removed, system-related reasons require active health policy responses, which should be multidimensional and differentiated so as to account for the fact that barriers to health care are unlikely to be uniform across groups of people and geographical areas. In this respect, decentralization of health care responsibilities may help to better target resources to population needs and to ensure more effective policy interventions.

As for the specific case of Italy, the absolute predominance of cost-related unmet health needs in the South claims for limiting out of pockets payments by patients. This can be done by either reducing co-payments and providing more extensive exemptions or enhancing the quality of public health services, so as to discourage individuals from choosing private services. It is worth noting, that health policies in Italy are, at the moment, following the opposite way: regions with large health care deficits, mostly in the South, have the statutory mandate to increase patient co-payments in order to curb expenditures. There is also evidence [29] that in these regions quality of health care services, at least as perceived by individuals, is worsening. Hence, efficiency improving policies seem to conflict with equity interventions. Concerning the rest of Italy, relatively more emphasis should be placed by regional governments in reducing waiting times (especially, in the Centre) and in providing support services, which could help people to fulfil their work and family commitments.

There are a number of limitations of this paper. First, as information on unmet health needs is self-reported, some concern could stem from the possibility of unreliable recall. On the contrary, given the specific study purpose, recognition errors due to non-clinically validated data are not a problem. Second, EU-SILC does not enable to distinguish between different experiences of self-perceived unmet health care needs. Specifically, it is not possible to discern situations in which people do not receive health care at all from situations in which they do not receive it in the way they want (e.g. in a timely manner). This fact limits the interpretation of the data, particularly in relation to specific policy options that might be considered to reduce the occurrence of unmet needs. Third, as already mentioned, unperceived unmet needs are completely neglected by the survey. Last but not least, the EU-SILC design is cross-sectional, and, thus, data on outcomes and determinants are collected simultaneously. Because of this, associations observed between variables cannot be inferred to be causal. In particular, the direction of causality can be confounded. This could be the case for the relationship between unmet health needs and the health status variables, which could suffer from reverse causality: unmet needs could exacerbate health conditions and so self-reported health status. In this regard, future research should focus on panel data based on different waves of EU-SILC to better disentangle the role played by each determinant in explaining unmet health needs.

## Endnotes

^1^ A part from dental care for which a similarly worded question is addressed in the EU-SILC, the way in which the present question is phrased in Italian could potentially omit certain kinds of health needs. Indeed, each European member state is quite free to decide how to word this question, though within the general framework provided by the EUROSTAT in the EU-SILC technical guidance. Compared to other countries that have opted for a more general and inclusive formulation, the Italian question focuses primarily on specialist care, even if the expression “medical treatment” sounds as quite generic and omni-comprehensive in Italian. This choice is probably due to the fact that in Italy access to GPs is open and free and, thus, it is mainly at the stage of access to specialist examinations and treatments that restrictions show up. In any case, this potential bias is expected to be a serious problem in case of cross-country comparisons but is indeed a minor issue when assessing inter-country differences.

## Competing interests

The author declares that she has no competing interests.
